# Stress and prevalence of hearing problems in the Swedish working population

**DOI:** 10.1186/1471-2458-11-130

**Published:** 2011-02-23

**Authors:** Dan Hasson, Töres Theorell, Martin Benka Wallén, Constanze Leineweber, Barbara Canlon

**Affiliations:** 1 Karolinska Institutet, Department of Physiology and Pharmacology, 171 77 Stockholm, Sweden; 2 Stress Research Institute, Stockholm University, SE-106 91 Stockholm, Sweden

## Abstract

**Background:**

Current human and experimental studies are indicating an association between stress and hearing problems; however potential risk factors have not been established. Hearing problems are projected to become among the top ten disabilities according to the WHO in the near future. Therefore a better understanding of the relationships between stress and hearing is warranted. Here we describe the prevalence of two common hearing problems, i.e. hearing complaints and tinnitus, in relation to different work-and health-related stressors.

**Methods:**

A total of 18,734 individuals were invited to participate in the study, out of which 9,756 (52%) enrolled.

**Results:**

The results demonstrate a clear and mostly linear relationship between higher prevalence of hearing problems (tinnitus or hearing loss or both) and different stressors, e.g. occupational, poorer self-rated health, long-term illness, poorer sleep quality, and higher burnout scores.

**Conclusions:**

The present study unambiguously demonstrates associations between hearing problems and various stressors that have not been previously described for the auditory system. These findings will open new avenues for future investigations.

## Background

Hearing problems are the most common sensory deficit in human populations, with hearing loss alone affecting more than 250 million people worldwide [1]. In 2002, the WHO estimated hearing loss to be the 13^th ^most frequent burden of disease in medium- and high-income countries, and it is projected to become among the top ten by the year 2030 [2]. Most epidemiological studies report prevalence figures of 10-15% for both hearing loss [1,3] and tinnitus [4,5] respectively. These figures may however not be inferable to hearing problems in a broader sense, since the prevalence is most often only calculated for either hearing loss or tinnitus. Consequently, a large Swedish study has recently addressed both issues concurrently, finding that approximately 32% of the Swedish working population suffer from either hearing complaints, tinnitus or both [6]. The study was also the first to provide evidence for a negative association between self-rated socioeconomic status (SES) and prevalence of hearing problems. It is well established that lower SES is associated with higher stress levels [7-9], so this finding may indirectly imply a relationship between stress and hearing problems.

While the deleterious effects of mechanical stress (i.e. noise) on hearing have been studied extensively in both animal models [10] and human populations [11,12], the notion of emotional stress as a modulator of the auditory system is rather novel. A complex set of pathways of the stress response have been identified, involving both sympathetic stimulation of adrenergic α-receptors within the cochlea [13,14], as well as neuro-endocrine responses primarily aimed at engaging the hypothalamic-pituitary-adrenal (HPA) axis [15]. Current research suggests that acute stress may protect the cochlea [16-19], whereas chronic stress exposure seems to be harmful to hearing [20]. The importance of a normal functioning of the HPA-axis for healthy hearing is supported by clinical studies showing that patients with tinnitus display signs of an impaired HPA-axis along with a higher degree of perceived stress, compared to non-tinnitus patients [21-23]. Additionally, Hasson et al. [24] recently found that symphony orchestra musicians with hearing problems exhibited lower heart rate variability (high frequency power), indicating an impaired ability to "unwind" and activate the parasympathetic system.

Human and animal studies are providing more evidence of an association between stress and hearing problems. It also needs be considered that long-term hearing problems can be stressful. Regarding the high prevalence of hearing problems in human populations and the negative future projections of the WHO, further investigations of the relationship between stress and hearing problems are warranted. Therefore, the aim of this study is to assess the prevalence of two common hearing problems, i.e. hearing complaints and tinnitus, in relation to different work- and health-related stressors. More specifically, we will study if prevalence differs with poor self-rated health, poorer sleep, higher burnout scores (work-related), and more symptoms of long-term stress and higher levels of performance-based self-esteem.

## Methods

### Population

The Swedish Work Environment Survey (SWES) is conducted biennially by Statistics Sweden (SCB) and consists of subsamples of gainfully employed people, aged 16-64 years, from the Labor Force Survey (LSF). These individuals were first sampled into the LFS through stratification by county of birth, sex, citizenship, and inferred employment status. The respondents to SWES 2003 and 2005 were invited to enroll in the Swedish Longitudinal Occupational Survey of Health (SLOSH) [25], which was initiated by the Stress Research Institute in 2006. The second data collection was conducted in April 2008 by Statistics Sweden, on behalf of the Stress Research Institute at Stockholm University. A total of 18,734 individuals were mailed self-completion questionnaires in 2008, out of which 9,756 (52%) working individuals responded. The total response rate of the study was however 11,441 (61%), including non-working participants (not analyzed in the present study). More detailed information about the cohort, response rate and characteristics of responders vs. non-responders has been published elsewhere [6]. There was no difference between responders and non-responders with regard to county of birth and citizenship.

### Questionnaire

Apart from socioeconomic status and demographic factors, it also included approximately 120 questions about psychosocial and physical work-environment, lifestyle, as well as physical and mental health.

Hearing problems were assessed with three questions. *Tinnitus*. Have you during the most recent time experienced sound in any of the ears, without there being an external source (so-called tinnitus) lasting more than five minutes? (No, Yes sometimes, Yes often, Yes all the time). *Tinnitus severity*. How much do you feel that the tinnitus sounds worry, bother or upset you? (Not at all, A little, Moderately, Severely). The questions about tinnitus were adapted from Davis [26] and Palmer et al. [27]. *Hearing complaints*. How difficult is it for you to (without hearing aid) hear what is said in a conversation between several persons? (Not difficult at all, Not very difficult, Quite difficult, Very difficult). In this study, hearing complaints reflects difficulties in communicating. The questions about hearing complaints were derived and adapted from Statistics Sweden and have been used in population studies for several years.

A new variable, "hearing problems", was computed based on the existence or non-existence of either tinnitus or hearing complaints or both. This consequently yielded three groups; those without hearing problems, those with either tinnitus or hearing complaints or those suffering from both. The cut-off for tinnitus was "yes, sometimes" or more often, and for hearing complaints "quite difficult" or "very difficult".

*Work-related stressors/threats*. Risks of being moved to another work/job against ones will, threats of getting fired were derived from the Swedish Labor Force Survey (LFS). Threats of bankruptcy were constructed for SLOSH 2008 to assess a threat particularly important for self-employed, a group who contacted the research group and expressed feelings of neglect in the SLOSH 2006 survey. The question was formulated: "Are you subjected to any of the following risks or threats in your work?" Response alternatives were yes/no.

*Self-rated health *was assessed with the single item "How would you rate your general state of health?" This question has been widely used in research [28-30] and the respondents answered on a Likert scale from ranging from 1 (very poor) to 5 (very good). Since only few participants had very poor SRH the categories quite poor and very poor were merged in the analyses.

*Long-term illness, inconvenience after an accident, any handicap or other weakness*. One question was asked about long-term illness, inconvenience after an accident or other weakness: "Do you have any prolonged sickness, accident-related complaints, a disability or other weakness?" This question was derived from the WOLFF (WOrk, Lipids and Fibrinogen-follow-up) [31,32] questionnaire and response alternatives were yes/no.

*Sleep quality*. Sleep quality was assessed by the single item: "How is your sleep quality in general?" This item was derived from the Karolinska Sleep Questionnaire [33]. Response ranged from 1 (very poor) to 5 (very good) on a 5-graded Likert scale.

*Burnout *was assessed with the Maslach Burnout Inventory general survey (MBI-GS) using the emotional exhaustion subscale [34]. The scale consists of five items, derived from the Maslach Burnout Inventory human services survey (MBI-HSS) in unmodified form. Scorings reach from 1 (every day) to 6 (a few times a year or less/never). Cronbach's alpha and stability for the subscale have been reported to be satisfactory. Strong support for the construct validity of the Swedish translation of the MBI-HSS has been found [35]. The index was calculated on the basis that 4 out of 5 items had to be answered in order be included in the index.

*Long lasting stress *(LLS) was assessed with 11 items reflecting stress arousal symptoms but not stress reactions. The participants were asked how they felt during the last three months with regard to both physiological (e.g. "I sweat easily even though I do not exert myself physically") and cognitive-behavioral symptoms (e.g. "I have worrying thoughts"; "I often feel tense"). The four response alternatives reached from "Not at all" to "Nearly all the time". The scale was introduced in the 2008 SLOSH questionnaire and is currently being validated (data not yet published). A factor analysis yielded one factor of interest. This factor included 7 of the 11 items and only the cognitive-behavioral symptoms. Factor loadings ranged from .675 ± 798 and a Chronbach's α of .863. The 7 included items were (including factor loading, FL):

A) I have days when I feel geared up all the time (FL = .675). B) I have days when I feel very pressured, on the verge of what I can handle (FL = .737). C) I find it hard to relax during my leisure time (FL = .787). D) I often feel tense (FL = .798). E) I often have disturbing thoughts (FL = .768). F) I often feel restless (FL = .720). G) I do not feel rested after taking it easy for a few days (FL = .699). The correlation between LLS and MBI-GS is r = .64, p < .0001, two-tailed. The index was calculated on the basis that 6 out of 7 items had to be answered in order be included in the index.

*Performance based self-esteem *[36,37] was assessed with four items (e.g. "At times, I have to be better than others to be good enough myself") with five response alternatives with the end-point labels "Fully disagree" to "Fully agree". Cronbach's alpha is between .85 and .89 and the one-year stability has been found to be satisfying. The index was calculated on the basis that 3 out of 4 items had to be answered in order be included in the index.

### Statistical analyses

The programs SPSS 18.0 and SAS 9.2 were used for statistical analyses. Prevalence was calculated via frequency plots and crosstabs were used for calculation of χ, Kendall's tau-b, and specific prevalence within different groups. Kendall's tau-b is a correlation analysis that illustrates the direction and magnitude of association between two variables. Multivariate analyses, proportional odds model (also called ordered logistic regression), were used to calculate possible interacting or confounding effects of age, gender and SES. Comparisons were made between those having no hearing problems compared to those with either tinnitus or hearing loss or both tinnitus and hearing loss. Statistical significance was set at p < 0.05 level.

### Ethical approval

The regional ethics committee in Stockholm approved the research project and all participants gave their informed consent to participate.

## Results

In a previous analysis of the present data it was shown that 31% of the working population report either hearing complaints or tinnitus (i.e. 25%) or both (i.e. 6%) and the prevalence of hearing problems increased with age, was higher among men and in persons with lower self-rated SES, and co-varied with exposure to noise at work [6]. In light of this background we now present the association between work- and health-related stressors and hearing problems.

Overall, the results describe an association between hearing problems and work- and health-related stressors. All the results were controlled for possible confounding effect of age, gender or SES with multivariate analyses. These analyses showed no confounding effect of these variables. The demographics of the population in the present study are as follows: 4,462 (46%) men and 5,294 (54%) women. The mean age was 48.6 (± 10.8) for men and 48.2 (± 10.5) for women. Age distribution was: under 40 years 1,148 (26%) men and 1,314 (25%) women; 41 ± 51 years 1,202 (27%) men and 1,520 (29%) women; 51-60 years 1,415 (32%) men and 1,754 (33%) women; 60 years or older 697 (26%) men and 706 (13%) women. Marital status: married 2,491 (56%) men and 2,946 (56%) women; unmarried 1,494 (34%) men and 1,516 (29%) women; divorced 444 (10%) men and 726 (14%) women; widow 33 (1%) and 106 (2%) women. With regard to highest completed educational level, 1,016 (10%) had no gymnasium, 4,510 (46%) had gymnasium, 619 (6%) had undergraduate studies of two years or less, 3,472 (36%) had undergraduate studies of three years or more and 134 (1%) hade post graduate studies.

### Work-related stressors/threats

Table 1 illustrates the prevalence of hearing problems in relation to different work-related stressors. There was a statistically significant difference in the prevalence of hearing problems between those who were and were not exposed to work-related stressors or threats such as risk of being moved to another work/job against ones will (χ^2 ^= 54.704_df = 2_, p < 0.0001) and threats of getting fired (χ^2 ^= 27.095_df = 2_, p < 0.0001). There was however no statistically significant difference between those who were exposed to threats of bankruptcy compared to those who were not.

**Table 1:** Prevalence of hearing problems in relation to different work-related stressors.

Employment-related threats	Prevalence of hearing problems			Relationship between threats and hearing problems; Kendall's τ-b and p-value
		
	No problems N (row %)	Either tinnitus or hearing loss N (row %)	Both tinnitus and hearing loss N (row %)	
**Risk of being moved to another work/job against ones will (N = 9,054)**.				0.076 p < 0.0001

Yes	997 (61)	514 (31)	126 (8)	
No	5,209 (70)	1,801 (24)	407 (6)	
				
**Threats of getting fired (N = 8,616)**.				0.055 p < 0.001

Yes	714 (62)	345 (30)	85 (7)	
No	5,232 (70)	1,818 (24)	422 (6)	
				
**Threats of bankruptcy (N = 8,385)**				0.018 p = 0.105

Yes	247 (65)	106 (28)	25 (7)	
No	5,556 (69)	1,999 (25)	452 (6)	

The proportional odds model did not exhibit any differences in odds ratios when age and socioeconomic status were included in models for neither women nor men. For instance, the unadjusted odds ratio of having hearing problems when exposed to a threat of being moved to another job against ones will (yes vs. no) were 1.39 for men (p < 0.001) compared to the adjusted odds ratios which were 1.43 (p < 0.001). For women, the corresponding values were 1.7 (p < 0.001) and when adjusted the value was 1.74 (p < 0.001).

### Self-rated health

**Figure 1:**
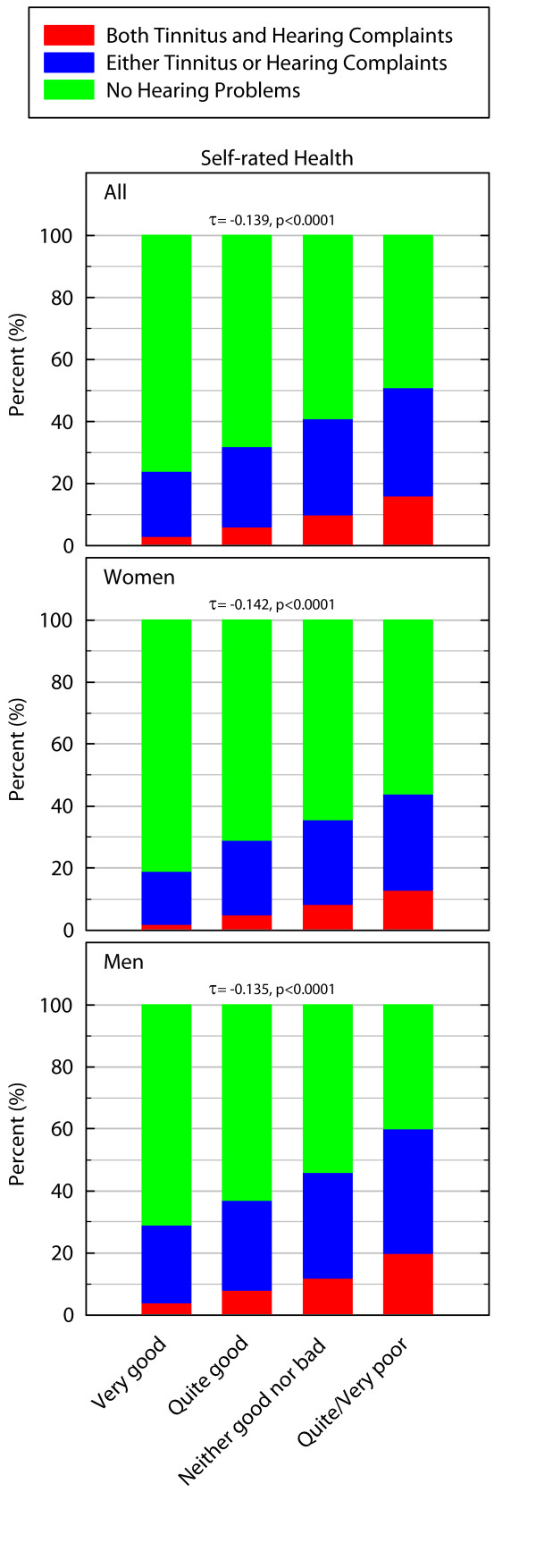
**Prevalence of hearing problems in percent in relation to different ratings of SRH**. The Kendall's τ-b value is indicated by a "τ".

There was a statistically significant difference in the prevalence of hearing problems between individuals with different levels of self-rated health (for all: χ^2 ^= 262.522_df = 6_, p < 0.0001, for women χ^2 ^= 141.535_df = 6_, p < 0.0001, for men: χ^2 ^= 123.306_df = 6_, p < 0.0001). Figure 1 clearly demonstrates that poorer SRH is associated with a higher prevalence of hearing problems. The association is negative (for all: Kendall's τ-b = -0.139 p < 0.0001, for women: Kendall's τ-b = -0.142 p < 0.0001, for men: Kendall's τ-b = -0.135 p < 0.0001) and indicates increasing prevalence of hearing problems among those with poorer health.

The proportional odds model did not exhibit any differences in odds ratios when age and socioeconomic status were included in models for neither women nor men. The unadjusted odds ratio of having hearing problems when reporting poor vs. good SRH were 3.89 for men (p < 0.001) compared to the adjusted odds ratios which were 3.29 (p < 0.001). For women, the corresponding values were 3.81 (p < 0.001) and when adjusted the value was 3.49 (p < 0.001).

### Long-term illness, inconvenience after an accident, any handicap or other weakness

There was a statistically significant difference in the prevalence of hearing problems between individuals with different handicaps and those without (χ^2 ^= 181.650_df = 2_, p < 0.0001). Those with long-term handicaps and illnesses reported more hearing problems (Table 2).

**Table 2:** Prevalence of hearing problems in relation to long-term illness, inconvenience after an accident, any handicap or other weakness.

Long-term illness, inconvenience after an accident, any handicap or other weakness	Prevalence of hearing problems			Relationship between long-term illness and hearing problems; Kendall's tau-b and p-value
		
	No problems N (row %)	Either tinnitus or hearing loss N (row %)	Both tinnitus and hearing loss N (row %)	
**Long-term handicaps**				0.120 p < 0.0001

Yes	1,268 (60)	640 (30)	239 (11)	
No	5,174 (72)	1,743 (24)	323 (5)	

The proportional odds model did not exhibit any differences in odds ratios when age and socioeconomic status were included in models for neither women nor men. For instance, the unadjusted odds ratio of having hearing problems after long-term illness (yes vs. no) were 1.92 for men (p < 0.001) compared to the adjusted odds ratios which were 1.76 (p < 0.001). For women, the corresponding values were 1.72 (p < 0.001) and when adjusted the value was 1.64 (p < 0.001).

### Sleep quality

**Figure 2:**
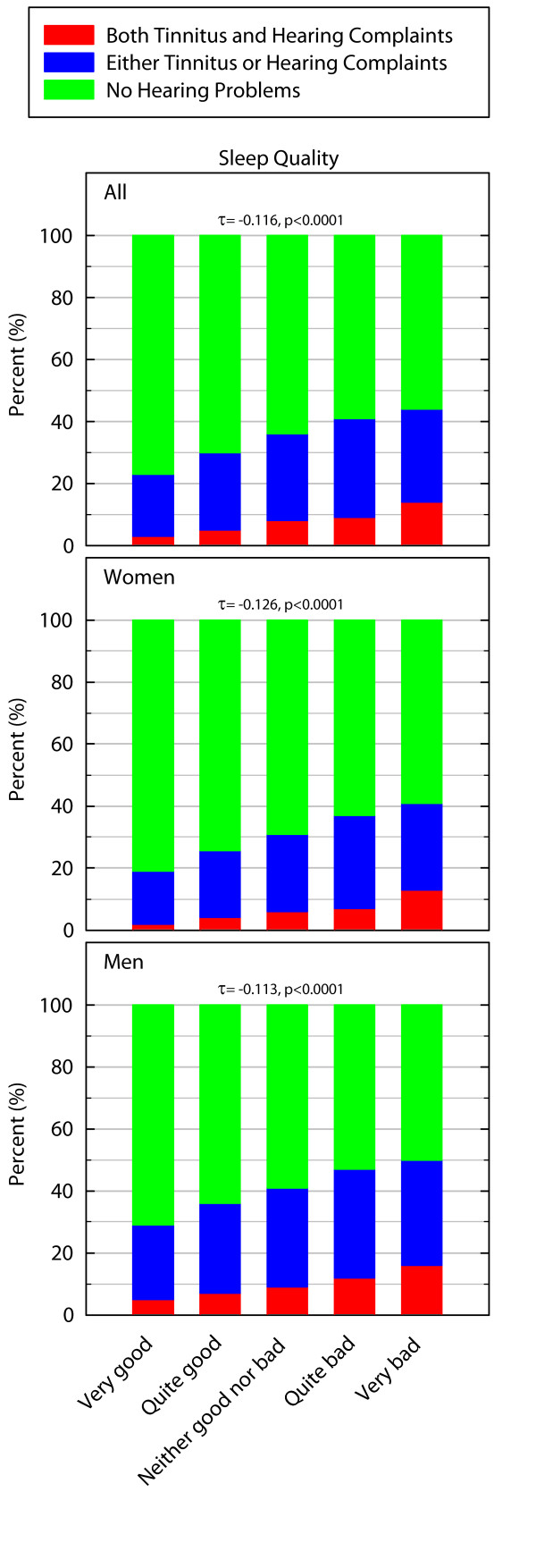
**Prevalence of hearing problems in percent in relation to different ratings of sleep quality**. The Kendall's τ-b value is indicated by a "τ".

There was a statistically significant difference in the prevalence of hearing problems between individuals with different levels of sleep quality (for all: χ^2 ^= 169.875_df = 8_, p < 0.0001, for women χ^2 ^= 112.509_df = 8_, p < 0.0001, for men: χ^2 ^= 75.068_df = 8_, p < 0.0001). The association was negative (for all: Kendall's τ-b = -0.116 p < 0.0001, for women: Kendall's τ-b = -0.126 p < 0.0001, for men: Kendall's τ-b = -0.113 p < 0.0001) and Figure 2 demonstrates that poorer sleep quality is associated with a higher prevalence of hearing problems. As tinnitus and hearing complaints may differ with regard to sleep, both of these variables were also analyzed separately. The prevalence of sleeping problems was significantly higher among those reporting tinnitus (χ^2 ^= 126.884_df = 4_, p < 0.0001, Kendall's τ-b = -0.106 p < 0.0001) compared to those reporting hearing complaints (χ^2 ^= 76.145_df = 4_, p < 0.0001, Kendall's τ-b = -0.081 p < 0.0001, see Table 3) and the associations were negative for both.

**Table 3:** Prevalence of sleeping problems among those with tinnitus and hearing loss respectively.

	**Relationship between sleep quality and those reporting tinnitus and hearing loss respectively**.	
	
Sleep quality	Tinnitus N (row %)	Hearing loss N (row %)
*All*		

Very good	376 (19)	137 (7)

Quite good	1,100 (25)	458 (10)

Neither good nor bad	559 (30)	237 (13)

Quite bad	389 (35)	174 (16)

Very bad	77 (39)	37 (19)

Number of participants and row percent in parentheses showing increasing prevalence of tinnitus with poorer sleep.

The proportional odds model did not exhibit any differences in odds ratios when age and socioeconomic status were included in models for neither women nor men. When the unadjusted odds ratio of having hearing problems when reporting poor vs. good sleep quality were 2.74 for men (p < 0.001) compared to the adjusted odds ratios which were 2.67 (p < 0.001). For women, the corresponding values were 3.51 (p < 0.001) and when adjusted the value was 3.24 (p < 0.001).

### Burnout

**Figure 3:**
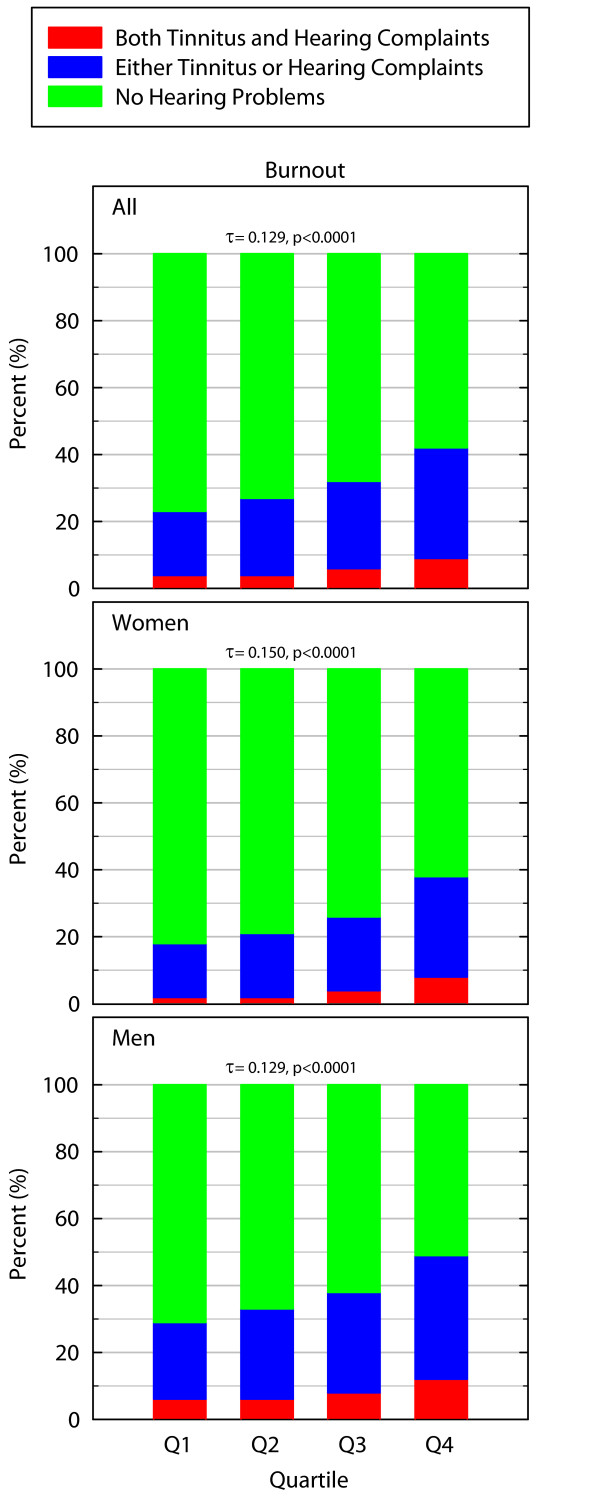
**Prevalence of hearing problems in percent in relation to different burnout scores (higher quartiles indicate more severe burnout symptoms)**. The Kendall's τ-b value is indicated by a "τ".

There was a statistically significant difference in the prevalence of hearing problems between those with higher burnout scores compared to those with lower scores (χ^2 ^= 214.473_df = 6_, p < 0.0001, for women χ^2 ^= 159.205_df = 6_, p < 0.0001, for men: χ^2 ^= 98.935_df = 6_, p < 0.0001). Hearing problems were significantly more prevalent among those with higher burnout scores. Multivariate analyses showed no age, gender or SES related differences in prevalence increases with increasing burnout scores. The association was positive (for all: Kendall's τ-b = 0.129 p < 0.0001, for women: Kendall's τ-b = 0.150 p < 0.0001, for men: Kendall's τ-b = 0.129 p < 0.0001) and Figure 3 demonstrates that higher burnout scores are associated with a higher prevalence of hearing problems.

The proportional odds model did not exhibit any differences in odds ratios when age and socioeconomic status were included in models for neither women nor men. For example, the unadjusted odds ratio of having hearing problems when being in the highest vs. lowest burnout quartile were 2.36 for men (p < 0.001) compared to the adjusted odds ratios which were 2.63 (p < 0.001). For women, the corresponding values were 2.80 (p < 0.001) and when adjusted the value was 2.79 (p < 0.001).

### Long-lasting stress

**Figure 4:**
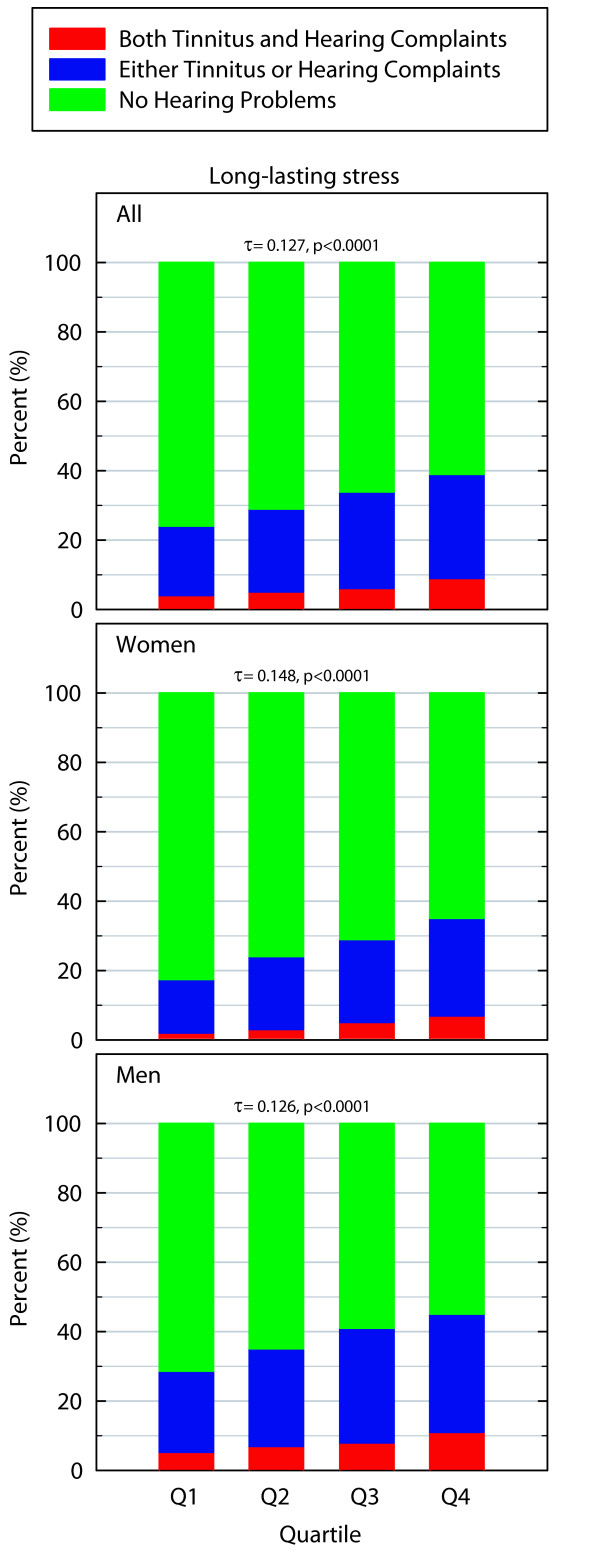
**Prevalence of hearing problems in percent in relation to symptoms of long-lasting stress (higher quartiles indicate more stress symptoms)**. The Kendall's τ-b value is indicated by a "τ".

There was a statistically significant difference in the prevalence of hearing problems between those with more symptoms of long-lasting stress scores compared to those with less (χ^2 ^= 196.855_df = 6_, p < 0.0001, for women χ^2 ^= 145.608_df = 6_, p < 0.0001, for men: χ^2 ^= 90.613_df = 6_, p < 0.0001). Hearing problems were more prevalent among those with more symptoms of long-lasting stress. Similarly to the pattern for burnout, the prevalence increase was higher for women than for men, even if it was less pronounced for this variable. The association was positive (for all: Kendall's τ-b = 0.127 p < 0.0001, for women: Kendall's τ-b = 0.148 p < 0.0001, for men: Kendall's τ-b = 0.126 p < 0.0001) and Figure 4 demonstrates that more symptoms of long-lasting stress are associated with a higher prevalence of hearing problems.

No major differences in odds ratios were found when age and socioeconomic status were included in models for women or men. The unadjusted odds ratio of having hearing problems when being in the highest vs. lowest long-lasting stress quartile were 2.06 for men (p < 0.001) compared to the adjusted odds ratios which were 2.42 (p < 0.001). For women, the corresponding values were 2.61 (p < 0.001) and when adjusted the value was 2.79 (p < 0.001).

### Performance-based self-esteem

**Figure 5:**
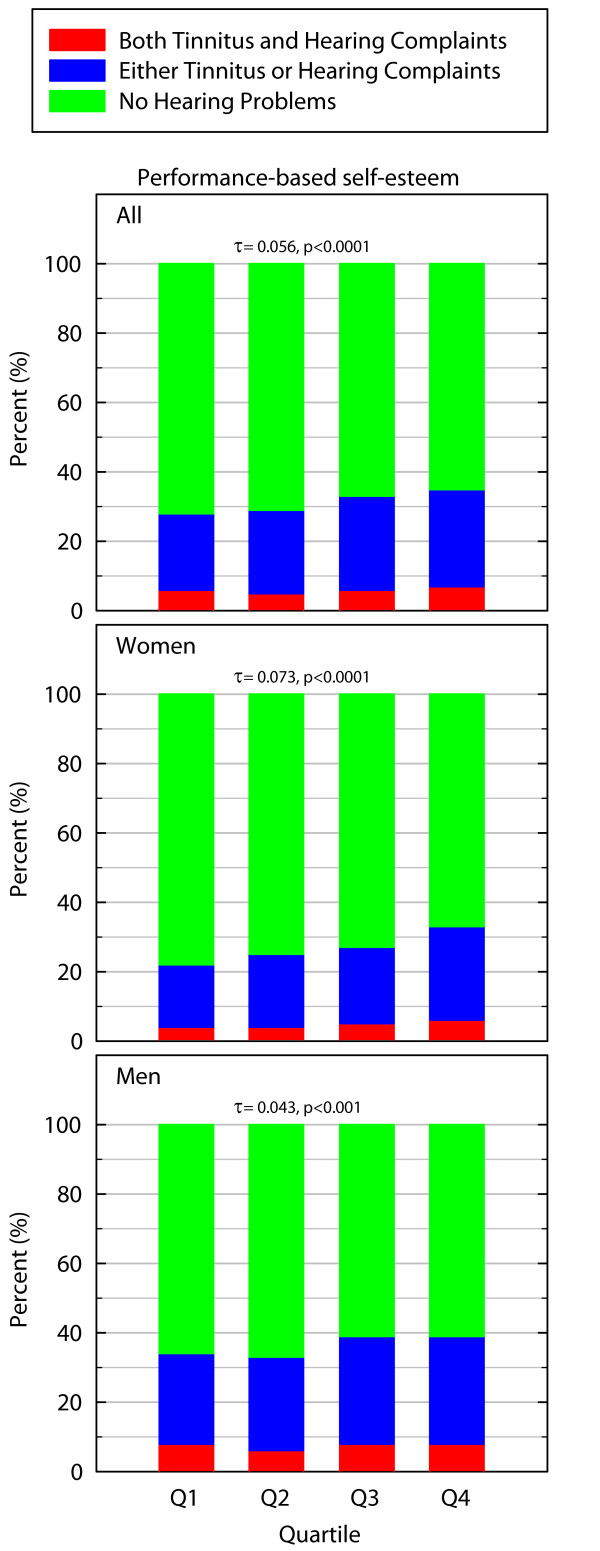
**Prevalence of hearing problems in percent in relation to performance-based self-esteem (higher quartiles indicate higher PBS)**. The Kendall's τ-b value is indicated by a "τ".

A statistically significant difference was found in the prevalence of hearing problems between those with higher and lower levels of PBS (χ^2 ^= 39.946_df = 6_, p < 0.0001, for women χ^2 ^= 36.410_df = 6_, p < 0.0001, for men: χ^2 ^= 15.181_df = 6_, p < 0.05). Hearing problems were more prevalent among those with higher levels of PBS. The association was positive and linear and more pronounced for women than for men (for all: Kendall's τ-b = 0.056 p < 0.0001, for women: Kendall's τ-b = 0.073 p < 0.0001, for men: Kendall's τ-b = 0.043 p < 0.01) and Figure 5 demonstrates that more symptoms of long-lasting stress are associated with a higher prevalence of hearing problems.

No major differences in odds ratios were found when age and socioeconomic status were included in models for women or men. The unadjusted odds ratio of having hearing problems when being in the highest vs. lowest PBS quartile were 1.26 for men (p < 0.001) compared to the adjusted odds ratios which were 1.41 (p < 0.001). For women, the corresponding values were 1.64 (p < 0.001) and when adjusted the value was 1.80 (p < 0.001).

## Discussion

The aim of the present study was to assess the prevalence of two common hearing problems, i.e. hearing complaints and tinnitus, in relation to different work-, life- and health-related stressors. The results demonstrate a clear and mostly linear relationship between higher prevalence of hearing problems (tinnitus or hearing complaints or both) and different stressors, symptoms of ill health and stress as well as poor sleep. Thus, the salient features of the present study illustrate that occupational stressors, poorer self-rated health, long-term illness, poorer sleep quality, higher burnout scores, more symptoms of long-lasting stress, and higher performance-based self-esteem are statistically significantly associated with a higher prevalence of hearing problems. To our knowledge, this is the first time that such vivid and systematic associations have been found for hearing problems (hearing complaints and tinnitus) in an extensive human population.

### Gender issues

The findings were consistent for both males and females, and men showed a slightly higher prevalence of hearing problems in absolute levels for all items analyzed except for performance-based self-esteem. Previously it has been found that the prevalence of hearing problems is higher among men and that prevalence increases with age [38]. The present findings elaborate on the previous findings and emphasize the correlation between poorer health, stress-related symptoms and hearing problems that affect men to a greater extent than women. Since hearing problems are becoming a major public health issue it is imperative to understand the factors that are directly and indirectly related to the underlying cause. The findings from the present study shed light on new attributes, biologically- and stress-related, that may be associated with the prevalence of hearing problems. The gender differences need to be interpreted in light of the fact that men and women may be exposed to different types of work environments and exposures that may affect the prevalence of hearing problems.

It is also noteworthy to mention that in the present study we have assessed two different factors for hearing problems. First we analyzed the prevalence of hearing problems either for tinnitus or hearing complaints and secondly for both tinnitus and hearing complaints. This assessment is more realistic since it is common for individuals who have hearing complaints to have tinnitus and vice versa. In a previous study, the prevalence of tinnitus was higher for men than women (5% vs. 3%, resp.)[6]. When analyzed for how often the tinnitus occurred, among those who answered that they suffer all the time, the prevalence for men was 10% vs. 5% for women. Interestingly, the effects of higher burnout scores, more symptoms of long-lasting stress and poor sleep quality also demonstrated a male dominance.

### Occupational consequences

Hearing problems were also more common among individuals exposed to occupational stressors, i.e. employment-related stress such as risk of being moved to another work/job against ones will and threats of getting fired. Threats of bankruptcy were not significantly associated with a higher prevalence of hearing problems. These problems include difficulties in communicating, lower awareness for important surrounding sounds such as telephone calls or warning signals or oversensitivity to sounds (hyperacusis) and even reduce productivity due to emotional exhaustion. These burdens become exaggerated for individuals with long-lasting stress symptoms and high burnout scores, as demonstrated in the present study. This is important for many reasons including the public awareness and the clarification that long-lasting stress and burnout symptoms are risk factors correlated with hearing problems.

### Self-rated health

Self-rated health (SRH) is one of the most widely used single measures of perceived current health status [28]. There is extensive evidence suggesting that SRH is a potent predictor of future mortality and morbidity [39,40], functional decline and disability, as well as the utilization of health care [39,41]. Most previous studies have shown that SRH is an independent predictor of future health outcomes, even after adjusting for self-ratings of other health-related measures, physician-reported health status, behavioral and psychosocial risk factors, socioeconomic status and environmental factors. Nevertheless, debate still continues about what SRH really represents [39,42,43].

It has been proposed that SRH represents an individual's general perception of health, including biological, psychological and social dimensions. Therefore SRH might be more sensitive in health monitoring than external measures of health [44]. Furthermore, it has been indicated that risk associated with poor SRH status is higher than that associated with poor objective health measures [45]. On the other hand, Kaplan & Camacho [46] found that objective health status has a stronger relationship with mortality than SRH.

A poorer self-rated health was correlated to hearing problems. Poor self-rated health can be influenced by several factors including social and marital status (Lindström, 2009), type of employment [47], sleep quality, sense of coherence, self-esteem, social support [28], higher cytokine levels [48], allostatic load [49] and unhealthy habits [50]. Unhealthy habits could include careless use of hearing protectors when in noisy environments or being exposed to excessive sound stimulation over long durations. Moreover, self-rated health is a reflection of the individual's quality of life and social abilities [51]. Depending on the severity of a particular hearing problem, consequent problems in communication and social interactions would result. This can cause problems in daily life as well as in the work environment. Since self-rated health is a compilation of biological, psychological and social assessments it may be a more accurate account of the consequences of hearing disabilities of the individual compared to, for example, pure tone audiometry.

### Sleep quality

Poor sleep quality was found to be associated with a higher prevalence of hearing problems in both men and women. In a study aimed at evaluating age specific prevalence of general symptoms it was found that five symptoms increased with increasing age [52]. These symptoms included insomnia, leg pain, joint pain, eye problems and impaired hearing. In another population study, patients with temporomandibular disorders were characterized for the auditory health [53]. The subjects who displayed auditory problems such as tinnitus, and perceived hearing complaints were found to be significantly more likely to consider themselves in poor health and have sleep disturbances than those without auditory problems. Two more studies have found correlations between sleep quality and hearing loss as well as tinnitus [54,55]. Thus, a correlation between poorer sleep quality and hearing problems is apparent in several different populations. In the present study, tinnitus was the more common disturbance when trying to sleep, probably since the perceived sound would increase annoyance and stress reactions. However, hearing complaints and tinnitus are often related co-morbid symptoms [38,56,57] and in the present study worse sleep quality was associated with higher prevalence of hearing complaints, albeit to a lesser extent than tinnitus.

In a previous study we found that individuals with hearing problems have a poorer ability to unwind or activate their parasympathetic system [24]. The present study confirms and strengthens this association since problems unwinding are strongly related to sleeping problems.

### Burnout and symptoms of long-lasting stress

It is by now well established that stress-related disorders, such as burnout, are associated with several forms of co-morbidity [58-62], e.g. long-term pain and psychiatric disorders. Now hearing problems may be added to the list. Our clinical experiences from a stress clinic shows that patients are often referred from audiologists. The present study showed clear associations between burnout as well as symptoms of long-lasting stress and hearing problems. Thus, hearing problems should be taken into account clinically in the diagnosis and treatment of stress-related disorders and vice versa. Furthermore, hearing problems were also more common among those with long-term illness, pains, inconveniences or handicaps. This strengthens the hypothesis about high levels of co-morbidity among individuals with hearing problems.

The relation between long-term stress and tinnitus has been previously explored and tinnitus sufferers often report enhanced problems by stress and fatigue [63]. It remains to be determined if tinnitus is a direct or indirect response of the auditory system to stress. Non-auditory-related brain regions, such as the limbic system have been shown to be activated during tinnitus [64]. The involvement of limbic areas during tinnitus offers a neuroanatomical correlate for stress-related tinnitus since the limbic region is a key region for regulating stress responses. Moreover, a major therapeutic strategy for tinnitus patients includes relaxation programs which have proven successful for many sufferers. Thus, taking into consideration the results of individuals with hearing problems (hearing complaints and tinnitus) of the present study and findings from prior tinnitus studies, it is becoming more apparent that stress can increase the prevalence of hearing problems. Furthermore, it has been shown that individuals with hearing problems have a worsened ability to unwind and activate the parasympathetic system [24].

### Performance-based self-esteem

In the present study there was a higher prevalence of hearing problems among those with higher PBS. High PBS is associated with high but vulnerable engagement, as well as higher prevalence of sickness presenteeism. When combined with high scores on a burnout scale it is a strong predictor of burnout and increased risk (OR = 2.84, CI 95% 1.61-5.01) for long-term sick leave [65]. This finding point to the direction that personality factors, especially PBS, may be important when assessing the risks for having hearing problems. It also indicates that hearing problems are multidimensional as they, apart from different stressors, also may be associated with other personality factors. These factors are by and of themselves complicated and multidimensional phenomena that have not yet been completely described. Therefore, more research should be directed at studying these interactions.

### Strengths and limitations

One of the major strengths with this study is the large sample size and that the sample is representative for the general Swedish working population. One weakness is the cross-sectional design, which does not allow conclusions about causality. Accordingly, prospective studies, which are forthcoming from our group, will be needed to illuminate the extent to which the observed associations are causal. Also, the study population is typical of a post-industrial country, with a high proportion of participants having a high educational level. It is possible that associations would be different in a population with a higher proportion of blue collar workers.

The assessment of hearing complaints and tinnitus via questionnaires has its advantages and disadvantages and there is no consensus as to which method is most valid. For example, the subjective evaluation of hearing problems may be difficult to interpret when there is a mild hearing loss. However, individuals having constant difficulties in understanding speech (especially in background noise) are usually well aware of their problem. It must be pointed out that an individual who has difficulties in understanding speech in background noise can have a completely normal audiogram. Thus, there are also limitations to audiological testing. This is not to say that an audiological test would not complement our subjective ratings, but we argue that addressing the question if there are "hearing problems" is acceptable. In fact, rating scales are commonly utilized to assess hearing problems [5,66] and it has been shown that the single question: 'Do you feel that you have a hearing loss?' was the most sensitive to assess hearing loss compared with pure-tone air conduction audiometry.

## Conclusion

In conclusion, the present study unambiguously demonstrates associations between hearing problems and occupational stressors, poorer self-rated health, higher burnout scores, more symptoms of long-lasting stress, long-term illness and poorer sleep quality. The interaction between the prevalence of hearing problems and the above mentioned features have not been previously described for the auditory system. These findings also indicate that hearing problems are multidimensional, which warrants further investigations of possible predictors.

## Competing interests

The authors declare that they have no competing interests.

## Authors' contributions

DH conducted the statistical analyses and drafted the manuscript together with BC, MBW, with substantial and essential input from CL and TT. TT was PI of the project and CL data manager. All authors have read and approved the final version of the manuscript.

## Pre-publication history

The pre-publication history for this paper can be accessed here:

http://www.biomedcentral.com/1471-2458/11/130/prepub
